# Effects on hip stress following sacroiliac joint fixation: A finite element study

**DOI:** 10.1002/jsp2.1067

**Published:** 2019-10-20

**Authors:** Amin Joukar, Ruchi D. Chande, R. Dana Carpenter, Derek P. Lindsey, Deniz U. Erbulut, Scott A. Yerby, Bradley Duhon, Vijay K. Goel

**Affiliations:** ^1^ Engineering Center for Orthopaedic Research Excellence (E‐CORE), Departments of Bioengineering and Orthopaedics The University of Toledo Toledo Ohio; ^2^ SI‐BONE Santa Clara California; ^3^ Department of Mechanical Engineering University of Colorado Denver Denver Colorado; ^4^ Department of Neurosurgery University of Colorado Denver Denver Colorado

**Keywords:** contact stress, finite element, hip, sacroiliac joint

## Abstract

For those patients who suffer from low back pain generated by the sacroiliac (SI) joint, fusion of the SI joint has proven to be an effective means of stabilizing it and reducing pain. Though it has shown promise, SI joint fusion raises clinical questions regarding its effect on neighboring joints such as the hip. As such, the purpose of this study was to determine the effects of SI joint fixation on the hip. A finite element spine‐sacroiliac‐hip (SSIH) model was developed and its functionality was verified against SI joint range of motion (ROM) and hip contact stress, respectively. The intact model was fixed in double leg stance at the distal femora, and loading was applied at the lumbar spine to simulate stance, flexion, extension, right and left lateral bending, and right and left axial rotation. Functionality was confirmed by measuring and comparing SI joint ROM and contact stress and area at the hip with data from the literature. Following verification of the intact SSIH model, both unilateral and bilateral SI joint fixation were modeled; hip contact stress and area were compared to the intact state. Average hip contact stress was ~2 MPa, with most motions resulting in changes less than 5% relative to intact; contact area changed less than 10% for any motion. Clinical significance: these results demonstrated that SI joint fixation with triangular titanium implants imparted little change in stress at the hip, which suggests that the risk of developing adjacent segment disease is likely low. Future clinical studies may be executed to confirm the results of this computational study.

## 1 INTRODUCTION

Low back pain (LBP) is the number one type of pain reported by adults and the leading cause of work‐related disability in the United States.^1^ Generators of LBP include the lumbar spine, hip, and sacroiliac (SI) joint, with the SI joint accounting for 15% to 30% of individuals with chronic low back pain.^1^, ^2^ The SI joint plays a key role in both load transfer and stability. It acts to transfer upper body weight through the pelvis and down into the lower extremities,^2^, ^3^ while its many undulations and surrounding ligaments aid in stabilizing the joint.^2^ Motions at the joint, although small, do occur, with the most motion occurring during flexion and extension of the sacrum relative to the ilia (often denoted as nutation and counter‐nutation, respectively^3^), followed by axial rotation and lateral bending.^4^ The joint, along with its surrounding tissues, may be disrupted by factors such as pregnancy or trauma, or it may suffer degeneration due to reasons of spine surgery, leg length discrepancies, or abnormal biomechanics.^2^, ^5^

To stabilize the joint and reduce pain, the SI joint has been fixed via both open and minimally invasive procedures,^6^ with solid triangular titanium implants being one such example of the latter.^4^, ^7^, ^8^ Clinical studies have proven SI joint stabilization with solid triangular implants to be an effective means of reducing pain, increasing quality of life, and reducing opioid use,^9^, ^10^, ^11^, ^12^ while computational and experimental biomechanics studies have demonstrated a reduction in range of motion (ROM) following placement of these triangular implants.^13^, ^14^

While fusion of the SI joint has shown promise, the use of triangular titanium implants to stabilize and fuse the SI joint raises clinical questions about how such fusion affects nearby joints. The phenomenon of adjacent segment disease (ASD) is well known in the spine; fusion of spinal motion segments has been correlated with additional stress on adjacent levels^14^, ^15^ including the SI joint.^16^ Using finite element analysis (FEA), Lindsey et al determined that stabilization of the SI joint via laterally‐placed triangular implants resulted in at least a 50% reduction in ROM at the SI joint but less than a 5% change in ROM at the lumbar spine. Though long term effects were yet unknown, the investigation showed little change in motion at the spine due to SI fusion, thereby indicating a lower potential to develop ASD.^14^ While this study investigated effects upon joints superior to the SI joint, research into the effects of SI joint fusion upon inferiorly located joints, specifically the hip, have yet to be explored.

The highly mobile hip joint is comprised of the acetabulum of the coxal bone and the femoral head; together their articulation, along with the surrounding soft tissues, facilitate both forward motion and simultaneous control of the body's center of gravity.^17^ The load‐bearing acetabulum and femoral head are mostly covered in hyaline cartilage of varying thicknesses and supported by the underlying subchondral and trabecular bony structures. Thicker sections of cartilage occur along the superior aspects of the articulating surfaces since these areas are prone to higher pressures during weight‐bearing.^17^, ^18^, ^19^ Consequently, changes in the distribution of weight along the femoral head surface can lead to atypical stress concentration in the hip cartilage and subsequent degeneration.^18^

The link between cartilage stress and degeneration and subsequent onset of disease like osteoarthritis^20^, ^21^ has prompted various studies to seek a better understanding of normal cartilage stress.^20^, ^21^, ^22^ Specifically, Anderson et al tested a cadaveric hip by simulating the reaction forces attained during walking, ascending, and descending stairs (motions investigated in Bergmann et al's study^23^) and measured the resulting contact pressures using pressure sensitive film.^22^ These data were then used to validate a patient‐specific, finite element model of contact pressure.^22^ Similarly, Henak et al studied contact pressure in the hip in a series of patient‐specific finite element models; each model was validated against a specimen, and additionally constitutive models and material definitions were evaluated.^21^ In both studies, the authors highlighted the use of such models to further study hip mechanics and the impacts of abnormal stresses on the joint.^21^, ^22^

Because degeneration of the hip, specifically the cartilage, may lead to disease, it is important to understand possible causes that may be attributable to this degeneration. Therefore, the current research sought to characterize hip stresses before and after fixation of the SI joint and assess the changes in contact stress with relation to future degeneration at the hip joint. This characterization was accomplished via a multipart finite element (FE) investigation: (a) a previously‐generated, intact lumbopelvic model was leveraged to validate a unilaterally‐treated SI joint model, (b) a hip model was generated and its function was validated against contact stress and contact area data from the literature, and (c) the two were incorporated into a single spine‐sacroiliac joint‐hip (SSIH) model to evaluate contact stresses at the hip due to uni‐ and bilateral SI joint fixation following applied loading.

## 2 METHODS

To characterize hip contact stresses following SI joint fusion, individual segment models (ie, lumbopelvic and hip) were first validated, and then these segments were combined into a single, intact SSIH model that was verified. Intact conditions were simulated prior to treated conditions, which incorporated uni‐or bilateral placement of three triangular titanium implants. In the following sections, steps taken to create the finite element segment models are described, followed by those taken to develop the combined model. Metrics for each part of the study are discussed.

### 2.1 FE segment model—Intact, treated SI joint, creation, and validation

With regard to the SI joint model, the current work leveraged a previously‐validated lumbopelvic model.^24^ This model is described in detail elsewhere,^24^ but briefly, it consisted of an intact, female lumbar spine (L1‐L5) with pelvis and fixed proximal femora whose functionality was validated against experimental SI joint range of motion (ROM) data.

To represent the treated SI joint model, the previously validated, intact pelvis model^24^ was modified to incorporate a unilateral SI joint treatment. Specifically, unilateral fixation was accomplished by laterally placing three triangular, titanium implants (SI‐BONE, Santa Clara, California) of lengths 60, 55, and 55 mm across a single SI joint. The implant's material property was that of Ti6Al4V (*E* = 115 GPa), a titanium alloy. The press‐fit of the triangular implants was defined as an interference fit of 0.2 mm, and a nonlinear surface‐surface contact with a frictional coefficient of 0.2 was assigned between the bone and implant.

To validate functionality of the treated SI joint, experimental conditions described by Lindsey et al^13^ were replicated in the computational space. The FE model was fixed in single leg stance and a 7.5 Nm moment was applied to the superior side of L1 to simulate the motions of flexion, extension, left and right lateral bending, and left and right axial rotation. The resulting rotations, calculated as the rotations of the sacrum minus those at the ilium, were measured for both left and right SI joints of the FE model and compared to the experimental data.

### 2.2 FE segment model—hip, creation, and validation

To create the hip joint, MIMICS 19 (Materialise, Leuven, Belgium) was used to segment femoral scans of a 55‐year‐old female cadaver and reconstruct the bony geometry, while Geomagic Studio 12 (Raindrop Geomagic Inc., 3D Systems, Morrisville, North Carolina) was used to smooth the femoral models. The femora were positioned relative to the pelvis per Wu et al by first defining the anatomical planes of each femur based on femoral bony features and the hip joint center of rotation, and then aligning these defined planes with the anatomical planes of the pelvis.^25^ Further, a scaling step was executed to appropriately position the femora relative to the acetabulae. As the femora originated from a second cadaver's scan, they required scaling such that (a) femoral head and acetabulum overlap would be avoided and (b) joint space between the two bony features would fall within the range of reported data (female right and left hip joint spaces of 3.43 mm ± 0.40 mm and 3.48 mm ± 0.68 mm, respectively).^26^ A sphere fit was performed on each femoral head and acetabulum, and the radii of the two fits were subtracted from one another to determine the joint space. Per the two scaling criteria mentioned earlier, this process was executed for scaling factors of 95% to 100% in 1% increments; 95% was determined to be the appropriate scaling factor (no overlap observed with right and left joint spaces of 3.46 and 3.36 mm, respectively).

Hypermesh 14 (Altair Engineering Inc., Troy, Michigan) was used to mesh the hip model; tetrahedral elements comprised the cancellous and cortical bone of the femora. A mesh convergence study was employed for the hip joint model in which seed sizes of 2, 3, 4, and 5 mm were applied to the femora, and functionality was assessed by monitoring the contact pressure during the simulated positions corresponding to walking, ascending stairs, and descending stairs. To simulate these positions, the femur was oriented relative to the pelvis as per the angles defined in Bergmann et al's data.^23^ Once the difference in contact pressure for any two consecutive seed sizes was within 2%, the coarser of the two meshes, in this case 3 mm, was selected and applied to the final femoral models.

Once the hip model was generated, finite element analysis of the hip was run in ABAQUS 6.14 (Dassault Systems, Providence, Rhode Island). Bony material properties were assigned per previous studies (Table 1). At the hip joint, a surface‐surface soft contact was defined between the femoral head and acetabulum to represent the cartilage. The soft contact was then assigned an exponential pressure‐overclosure behavior with contact pressure and overclosure defined as 50 MPa and 0.5 mm, respectively, in which stress at the hip joint would increase as the distance between the two contact surfaces (ie, femoral head and acetabulum) decreased.

**Table 1:** FE model material properties

Component	Material properties	Constitutive relation	Element type
Vertebral cortical bone^27^	*E* = 12 000 MPa *υ* = 0.3	Isotropic, elastic	8 Nodes brick element (C3D8)
Vertebral cancellous bone^27^	*E* = 100 MPa *υ* = 0.2	Isotropic, elastic	4 Nodes tetrahedral element (C3D4)
Pelvic cortical bone (Sacrum, Ilium)^14^	*E* = 17 000 MPa *υ* = 0.3	Isotropic, elastic	4 Nodes tetrahedral element (C3D4)
Sacrum cancellous bone^28^	Heterogeneous	Isotropic, elastic	4 Nodes tetrahedral element (C3D4)
Ilium cancellous bone^28^	*E* = 70 MPa *υ* = 0.2	Isotropic, elastic	4 Nodes tetrahedral element (C3D4)
Femur cortical bone^22^	*E* = 17 000 MPa *υ* = 0.29	Isotropic, elastic	4 Nodes tetrahedral element (C3D4)
Femur cancellous bone^22^	*E* = 100 MPa *υ* = 0.2	Isotropic, elastic	4 Nodes tetrahedral element (C3D4)
Ground substance of annulus fibrosis^29^	*C*_10_ = 0.035 *K*_1_ = 0.296 *K*_2_ = 65	Hyperelastic anisotropic (HGO)	8 Nodes brick element (C3D8)
Nucleus pulposus^27^	*E* = 1 MPa *υ* = 0.499	Isotropic, elastic	8 Nodes brick element (C3D8)
Anterior longitudinal^27^	7.8 MPa (<12%), 20 MPa (>12%)	Non‐linear hypoelastic	Truss element (T3D2)
Posterior longitudinal^27^	10 MPa (<11%), 20 MPa (>11%)	Non‐linear hypoelastic	Truss element (T3D2)
Ligamentum flavum^27^	15 MPa (<6.2%), 19.5 MPa (>6.2%)	Non‐linear hypoelastic	Truss element (T3D2)
Intertransverse^27^	10 MPa (<18%), 58.7 MPa (>18%)	Non‐linear hypoelastic	Truss element (T3D2)
Interspinous^27^	10 MPa (<14%), 11.6 MPa (>14%)	Non‐linear hypoelastic	Truss element (T3D2)
Supraspinous^27^	8 MPa (<20%), 15 MPa (>20%)	Non‐linear hypoelastic	Truss element (T3D2)
Capsular^27^	7.5 MPa (<25%), 32.9 MPa (>25%)	Non‐linear hypoelastic	Truss element (T3D2)
Anterior SIJ^30^	125 MPa (5%), 325 MPa (>10%), 316 MPa (>15%)	Non‐linear hypoelastic	Truss element (T3D2)
Short posterior SI^30^	43 MPa (5%), 113 MPa (>10%), 110 MPa (>15%)	Non‐linear hypoelastic	Truss element (T3D2)
Long posterior SI^30^	150 MPa (5%), 391 MPa (>10%), 381 MPa (>15%)	Non‐linear hypoelastic	Truss element (T3D2)
Intraosseus^30^	40 MPa (5%), 105 MPa (>10%), 102 MPa (>15%)	Non‐linear hypoelastic	Truss element (T3D2)
Sacrospinous^30^	304 MPa (5%), 792 MPa (>10%), 771 MPa (>15%)	Non‐linear hypoelastic	Truss element (T3D2)
Sacrotuberous^30^	326 MPa (5%), 848 MPa (>10%), 826 MPa (>15%)	Non‐linear hypoelastic	Truss element (T3D2)
Gluteus maximus^31^	*k* = 344 N/mm	—	Connector element
Gluteus medius^31^	*k* = 779 N/mm	—	Connector element
Gluteus minimus^31^	*k* = 660 N/mm	—	Connector element
Psoas major^31^	*k* = 100 N/mm	—	Connector element
Adductor magnus^31^	*k* = 257 N/mm	—	Connector element
Adductor longus^31^	*k* = 134 N/mm	—	Connector element
Adductor brevis^31^	*k* = 499 N/mm	—	Connector element

Hip joint functionality was validated against research conducted by Anderson et al^22^ and Harris et al.^20^ The experimental study simulated motions, specifically walking, ascending stairs, and descending stairs,^22^ described in an earlier instrumented hip clinical study by Bergmann et al.^23^ This study validated hip functionality by comparing hip contact stress and area against those determined by Anderson et al^22^ (described earlier) and Harris et al.^20^ Harris et al developed patient specific FE models based on volunteers' scans but the relative positions of the femoral heads and acetabulae of these models were driven per data from Bergmann et al; contact metrics produced by the models were measured and reported. The same boundary conditions and activities were simulated in the current work: the pelvis was constrained while the femur was positioned per the Bergmann angles corresponding to walking, ascending stairs, and descending stairs.^23^ Stress was calculated on a nodal basis with both peak and average von Mises stresses reported at the hip. As in the Anderson et al study, a threshold of 0.5 MPa was established^22^ such that contact stresses were recorded at only those nodes at or above the threshold during the stress calculation. Peak stress values for both the femur and acetabulum were obtained and averaged to obtain the peak contact stress acting at the hip joint. Contact area was measured on an elemental basis including elements exhibiting stresses at or above 0.5 MPa. Both stress and area were then evaluated against Anderson et al's and Harris et al's reported data.^20^, ^22^

### 2.3 SSIH FE model—intact, creation, and verification

**Figure 1:**
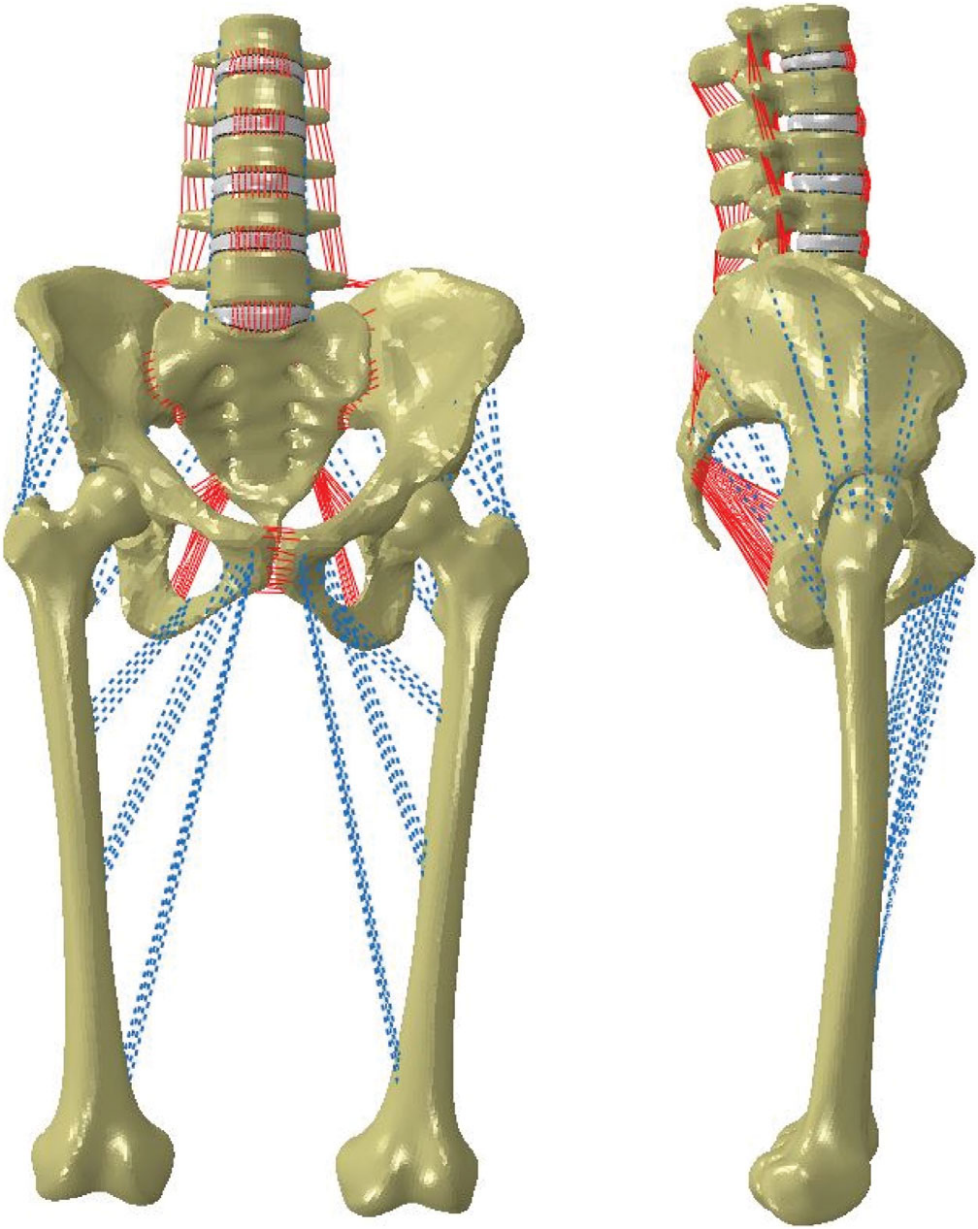
Finite element model of intact, combined lumbopelvic‐femora model. Coronal (left) and sagittal (right) views

Following validation of the individual hip model, the lumbopelvic model and femora were combined into a single, intact model (SSIH, Figure 1), and its functionality as a complete model was verified given new boundary conditions. Material properties of the individual models were carried over to the SSIH model along with a few additions. Specifically, muscles spanning the hip joint from their physiologic origins to insertions were included as connector elements with stiffnesses assigned per Phillips et al (Table 1).^31^ The SSIH model was assembled in ABAQUS 6.14, and the final model contained a total of 767 694 elements.

To reiterate, the focus of this portion of the study was to confirm the combined model's functionality given new boundary conditions. Here, the model maintained double leg stance as it was fixed at the femoral condyles; the remainder of the model was constrained anatomically via the soft tissue structures. Thus, a noteworthy distinction of this model is its inclusion of mobile hip joints, which, to the authors' knowledge, is a feature that has not been included with other models containing the spine, pelvis, and femora.

**Figure 2:**
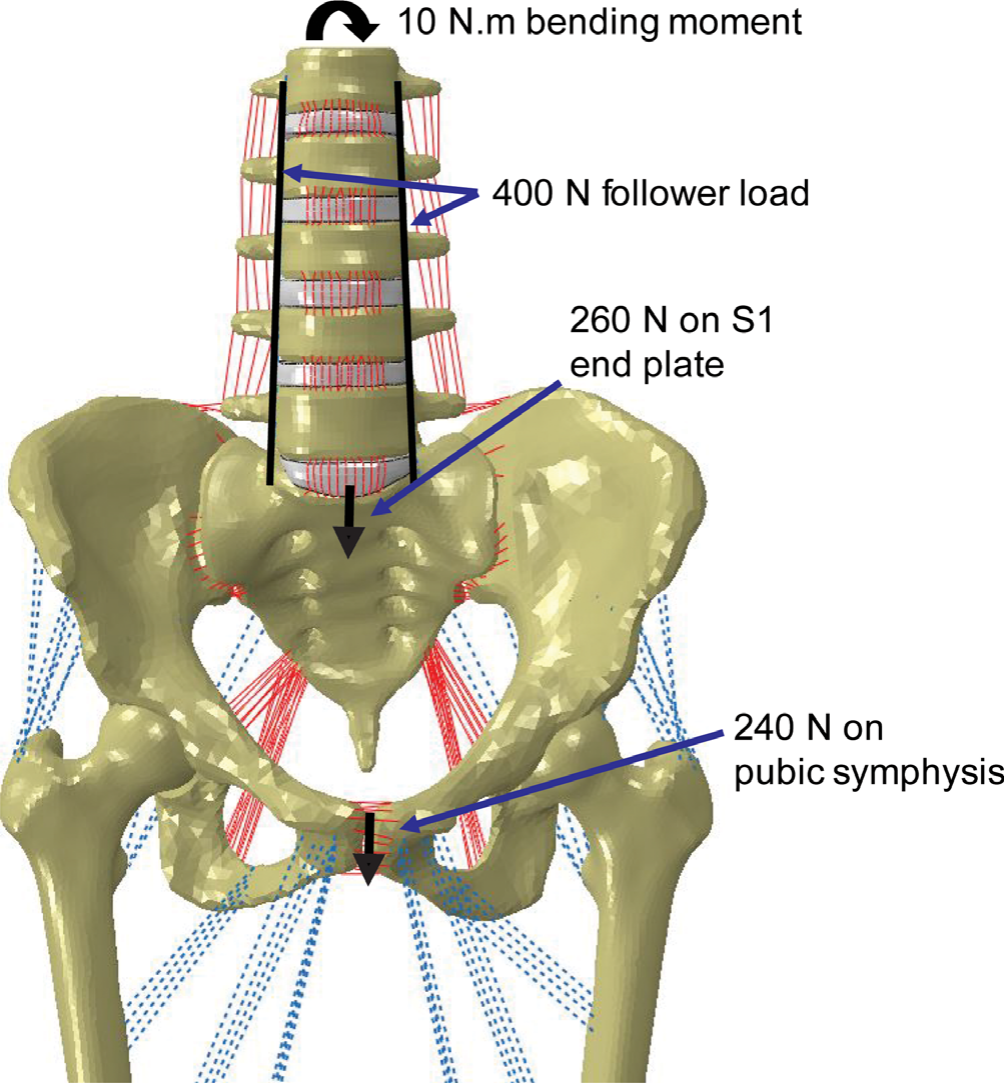
Loading conditions applied to the combined model. To simulate stance, a 400 N follower load and 500 N body weight were applied; a 10 Nm moment was added to simulate flexion/extension, left and right lateral bending, and left and right axial rotation

To substantiate the model's functionality in stance position, a force of 500 N was distributed between the sacral promontory and pubic tubercles to simulate torso weight, while a follower load of 400 N was used to stabilize the lumbar spine. The intact model was further subjected to motion in the three anatomical planes, specifically flexion/extension (F/E), left/right lateral bending (LB), left/right axial rotation (AR), by applying a 10 Nm moment to the superior endplate of L1 (Figure 2). For each motion, the SI joint range of motion (ROM), hip contact stress (average and peak), and hip contact area were calculated; this intact data served as the baseline against which the treated models were compared.

### 2.4 Treated SSIH FE model—Creation and functional comparisons against intact

**Figure 3:**
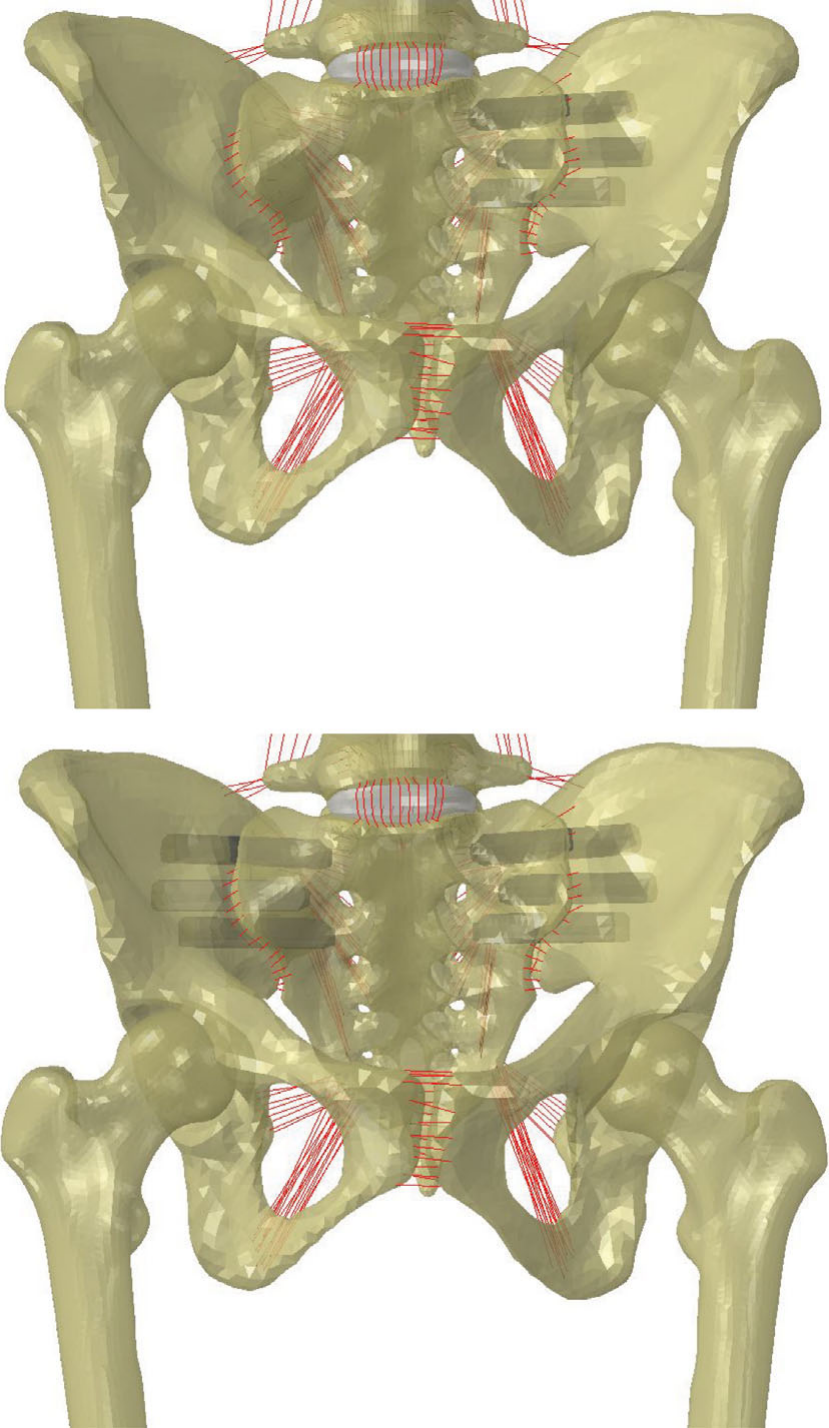
Spine‐sacroiliac‐hip model with fixation of sacroiliac joints via triangular titanium implants. (Top) Left‐unilateral treatment, (Bottom) bilateral treatment

Following all validation and verification activities, the SSIH model was treated to assess the effect of SIJ stabilization on the hip joint relative to the intact condition, specifically the stresses at the hip joint before and after stabilization. Here, a treated model refers to the combined model described above with one or more stabilized SI joints as previously described (Figure 3). Loading and boundary conditions, simulated motions, and observed metrics for the treated models were the same as for the intact SSIH model. Regarding the unilateral treatments, SI joint range of motion as well as hip stress and area were evaluated at both the ipsilateral and contralateral joints.

## 3 RESULTS

### 3.1 FE segment model—Treated SI joint

**Figure 4:**
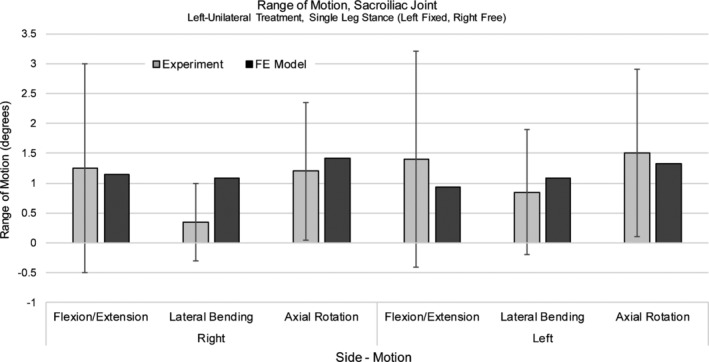
Sacroiliac joint range of motion, left‐unilateral treatment. FE model results were validated against Lindsey et al (Mean ± SD).^13^ Model was fixed in single leg stance (left leg fixed, right leg free)

The ROMs of the left and right SI joints ranged from approximately 1° to 1.5° for all motions (ie, flexion/extension, lateral bending, axial rotation), which compared favorably with published variability for the unilaterally treated SI joint model^13^ (Figure 4).

### 3.2 FE segment model—Hip validation

Contact stress was found to be similar in comparison to the data presented by Anderson et al (Table 2). Peak stresses were higher than those found in Anderson et al (Table 3), while contour maps revealed similar stress patterns as those seen in the validation reference.^22^

**Table 2:** Hip validation—contact stress, area

Motion	Average contact stress (MPa)							Contact area (mm^2^)						
	Femoral Head		Acetabulum		Anderson et al^22^		Harris et al^20^	Femoral head		Acetabulum		Anderson et al^22^		Harris et al^20^
	Right	Left	Right	Left	Experimental	FE	FE	Right	Left	Right	Left	Experimental	FE	FE
W	4.52	4.24	3.73	3.44	4.7	5.7	1.08 ± 0.32	552	514	1066	981	425.1	304.2	700 ± 150
AS	4.73	5.77	3.71	4.48	5.0	5.1	1.18 ± 0.27	544	395	980	836	321.9	366.1	690 ± 240
DS	4.04	4.82	3.53	4.04	4.4	6.2	1.23 ± 0.32	682	546	1280	998	375	325	730 ± 160

Abbreviations: AS, ascending stairs; DS, descending stairs; FE, FE results.^22^; W, walking.

**Table 3:** Hip validation, peak contact stress

	Peak contact stress (MPa)		
	Current study^a^		Anderson et al study^22^ (FE model)
Motion	Right	Left	
Walking	13.41	10.56	10.78
Ascending stairs	15.58	13.62	11.61
Descending stairs	13.82	13.30	12.73

a Peak stress, current study = average of peak stresses on femoral head, acetabulum; reported for right, left hips.

As for contact area, the values observed at the hip in the current study were higher than those found in the Anderson et al study^22^ (Table 2), but were more comparable to the higher values found in the Harris et al investigation (Table 2), a similar study to that of Anderson et al's in which multiple patient‐specific hip models were considered.^20^, ^22^

### 3.3 SSIH FE model—Intact

**Figure 5:**
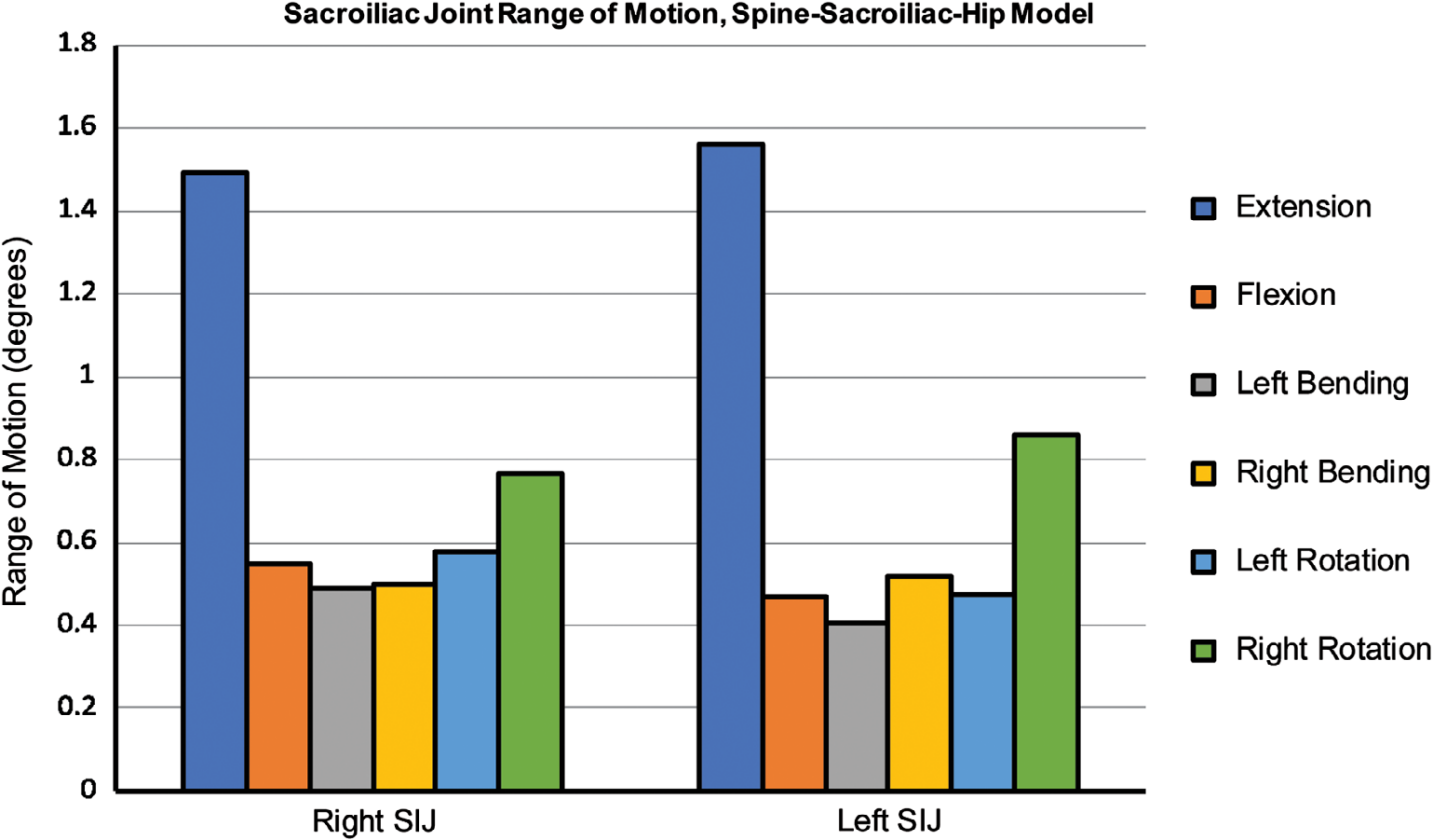
Sacroiliac joint range of motion for the intact, spine‐sacroiliac‐hip model. Ranges of motion (ROM) for the current model, which was fixed in double leg stance, were similar to those reported by Joukar et al^24^; measured ROMs were within the normal joint ROM

**Figure 6:**
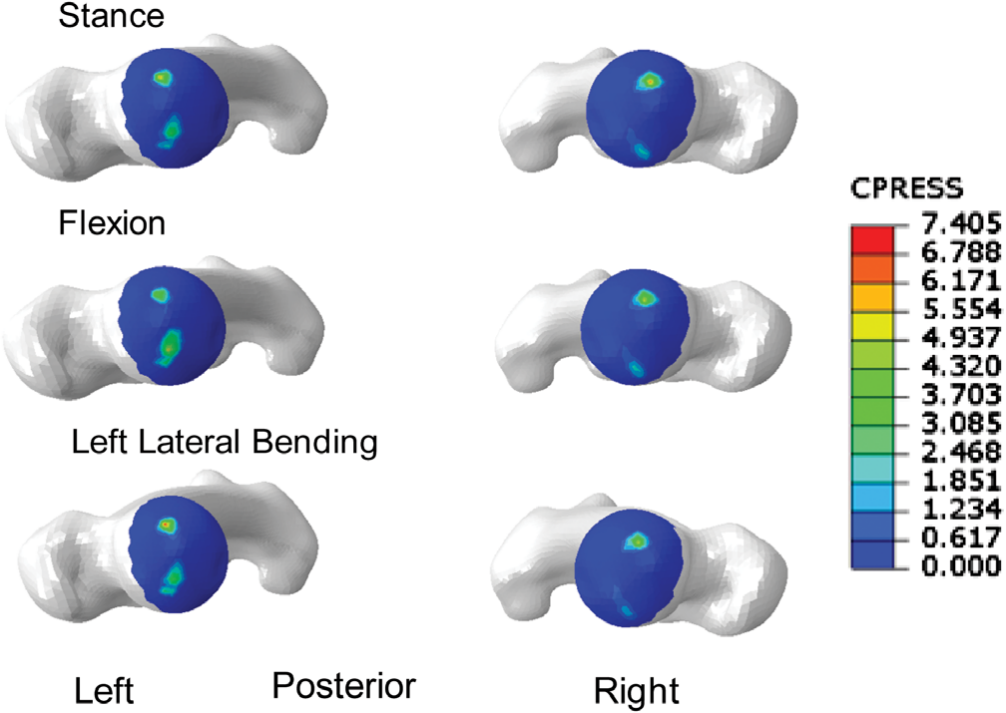
Representative stress contours for the intact, Lumbopelvic‐Femora model. Superior views of femoral heads in stance (top), flexion (middle), and left lateral bending (bottom)

SI joint range of motion for the current, double leg stance model (Figure 5) was less than that reported for single leg stance.^24^ Further, when compared to double leg stance data reported by Joukar et al,^24^ ROM for the right and left SI joints of the current model were consistent. Average and peak contact stresses at the hip (Table 4, Figure 6) were found to be less than those in the walking, ascending, and descending stairs models, though they were similar in magnitude to average stresses reported in Harris et al for various activities.^20^ Finally, contact areas were on the same order of magnitude as those found in the literature (Table 5).

**Table 4:** Combined model, average and peak contact stresses for right, left femoral heads

Motion	Average contact stress (MPa)		Peak contact stress (MPa)	
	Right	Left	Right	Left
Standing	1.81	1.87	6.20	5.96
Flexion	1.65	2.29	5.28	5.48
Extension	1.86	1.76	7.40	5.30
Right LB	1.94	1.62	7.10	4.80
Left LB	1.50	1.94	5.23	6.85
Right AR	1.66	1.94	6.10	6.00
Left AR	1.82	1.88	6.40	5.68

Abbreviations: AR, axial rotation; LB, lateral bending.

**Table 5:** Combined model, contact area for right, left femoral heads

Motion	Contact area (mm^2^)	
	Right	Left
Standing	224	207
Flexion	232	222
Extension	239	213
Right LB	238	201
Left LB	222	245
Right AR	224	207
Left AR	224	207

Abbreviations: AR, axial rotation; LB, lateral bending.

### 3.4 SSIH FE model—Comparison, intact vs treated

**Figure 7:**
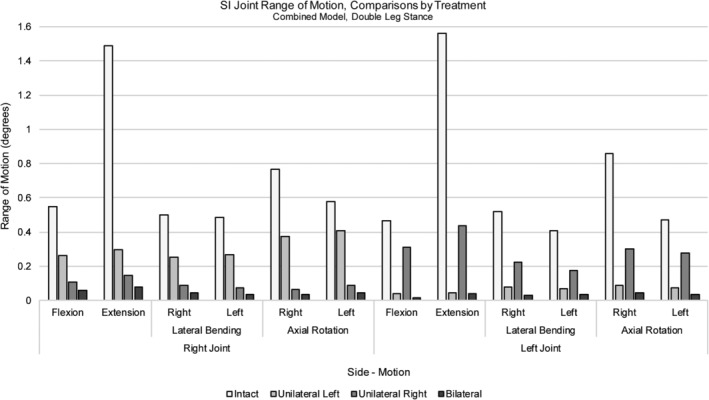
Sacroiliac joint range of motion, combined model. Comparisons between the intact, left‐, right‐, and bilaterally treated simulations for both right and left sacroiliac (SI) joints. Loading included body weight along with a 400 N follower load and 10 Nm moment; models were fixed at the femoral condyles in double leg stance

SI joint range of motion for the intact model following flexion/extension, left and right lateral bending, and left and right axial rotation is given alongside the unilaterally and bilaterally treated models in Figure 7. With regard to fixation of the SI joint, all treatments resulted in a reduction in motion of the fixated side(s) with the ROM of the bilateral treatment being less than that of the unilateral treatments. Further, when examining the treated cases, the unilaterally‐treated SI joint ROM was less than that of the contralateral (untreated) side. This was true regardless of whether the left or right SI joint was treated. When comparing the left and right unilateral treatments, the left SI joint tended to have higher ranges of motion than the right for each movement in this patient‐specific model.

**Figure 8:**
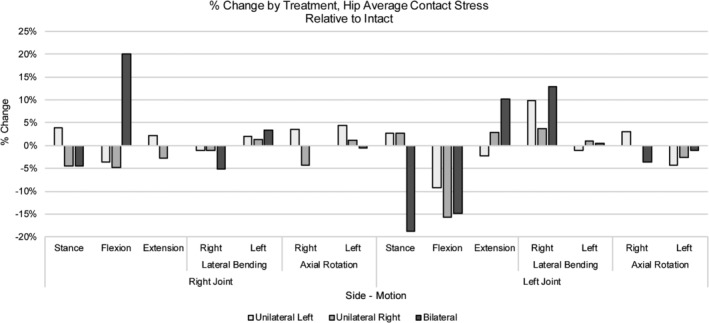
% Change by treatment, hip average contact stress for various loading configurations. Changes in average contact stress relative to intact are depicted by treatment for the left and right hip joints. Positive and negative values equate to an increase and decrease, respectively, from intact

**Figure 9:**
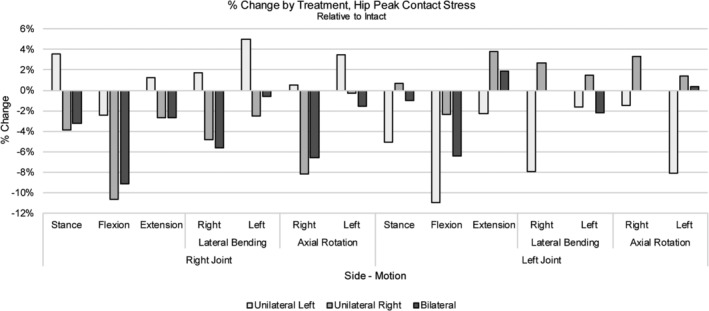
% Change by treatment, hip peak contact stress for various loading configurations. Changes in peak contact stress by treatment for the left and right hip joints are shown. Positive and negative values equate to an increase and decrease, respectively, from intact

The average contact stress in stance was 1.81 and 1.87 MPa for the right and left hip joints, respectively (Table 6), while peak contact pressures were approximately 6 MPa (Table 7). Following application of a 10 Nm moment, average stresses for all motions (ie, F/E, LB, AR) ranged between 1.5 and 1.98 MPa for the right hip and 1.62 and 2.29 MPa for the left hip (Table 6). Following unilateral or bilateral treatment, stress at the right hip joint exhibited changes no greater than approximately 5% relative to the intact condition after most motions (Figure 8). Only the bilaterally treated model demonstrated a greater change in the case of flexion; a 20% increase was noted though the magnitude of the resulting stress remained under 2 MPa. Relative to the intact model, the left hip demonstrated approximate increases in average stress of 10% and 13% in extension and right lateral bending, respectively; however, the magnitudes of these increases remained below 2 MPa. Other motions that resulted in changes greater than 10% relative to intact included stance (bilateral treatment) and flexion (right unilateral and bilateral treatments); these changes equated to decreases in average stress with magnitudes all below 2 MPa (Table 6, Figure 8). As for peak contact stress for either the right or left hip, both increases and decreases were observed, though the increases in peak stress were all below 5% (Figure 9).

**Table 6:** Average contact stress by treatment, right and left hip joints for various loading configurations

	Average contact stress (MPa)													
	Stance		Flexion		Extension		Right LB		Left LB		Right AR		Left AR	
Side	Right	Left	Right	Left	Right	Left	Right	Left	Right	Left	Right	Left	Right	Left
Intact	1.81	1.87	1.65	2.29	1.86	1.76	1.94	1.62	1.50	1.94	1.66	1.94	1.82	1.88
Unilateral—L	1.88	1.92	1.59	2.08	1.90	1.72	1.92	1.78	1.53	1.92	1.72	2.00	1.90	1.80
Unilateral—R	1.73	1.92	1.57	1.93	1.81	1.81	1.92	1.68	1.52	1.96	1.59	1.94	1.84	1.83
Bilateral	1.73	1.52	1.98	1.95	1.86	1.94	1.84	1.83	1.55	1.95	1.66	1.87	1.81	1.86

Abbreviations: AR, axial rotation; L, left; LB, lateral bending; R, right.

**Table 7:** Peak stress by treatment, right and left hip joints for various loading configurations

	Peak contact stress (MPa)													
	Stance		Flexion		Extension		Right LB		Left LB		Right AR		Left AR	
Side	Right	Left	Right	Left	Right	Left	Right	Left	Right	Left	Right	Left	Right	Left
Intact	6.20	5.96	5.28	5.48	7.40	5.30	7.10	4.80	5.23	6.85	6.10	6.00	6.40	5.68
Unilateral—L	6.42	5.66	5.15	4.88	7.49	5.18	7.22	4.42	5.49	6.74	6.13	5.91	6.62	5.22
Unilateral—R	5.96	6.00	4.72	5.35	7.20	5.50	6.76	4.93	5.10	6.95	5.60	6.20	6.38	5.76
Bilateral	6.00	5.90	4.80	5.13	7.20	5.40	6.70	4.80	5.20	6.70	5.70	6.00	6.30	5.70

Abbreviations: AR, axial rotation; L, left; LB, lateral bending; R, right.

**Figure 10:**
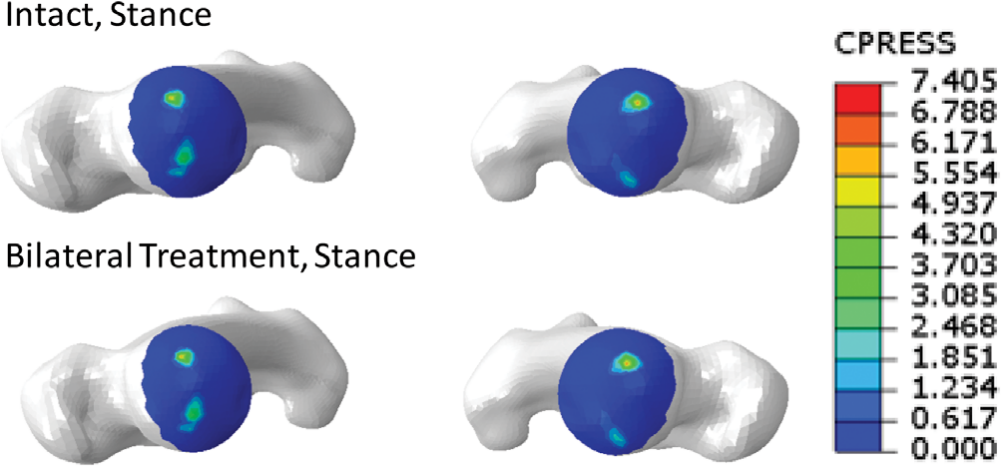
Representative stress contours for the intact and bilaterally treated SSIH models. Superior views of femoral heads in stance for the intact (top) and bilaterally‐treated (bottom) models

Notably, contact area in stance was higher for the right hip joint in comparison to the left; this was true for the intact model as well as across all treatments (Table 8). Hip contact area for both left and right hip joints changed less than 10% when compared to the intact case for any motion with various motions resulting in no change in contact area after treatment (Table 9). Stress patterns were similar among different treatments with two regions of contact located generally anteriorly and posteriorly (Figure 10).

**Table 8:** Contact area by treatment, right and left hip joints for various loading configurations

	Contact area (mm^2^)													
	Stance		Flexion		Extension		Right LB		Left LB		Right AR		Left AR	
Side	Right	Left	Right	Left	Right	Left	Right	Left	Right	Left	Right	Left	Right	Left
Intact	224	207	232	222	239	213	238	201	222	245	224	207	224	207
Unilateral—L	224	207	254	230	236	213	237	201	227	223	221	207	224	209
Unilateral—R	236	207	226	241	226	213	244	202	219	245	239	216	224	207
Bilateral	236	207	226	228	230	213	244	195	222	236	244	207	232	207

Abbreviations: AR, axial rotation; L, left; LB, lateral bending; R, right.

**Table 9:** % Change by treatment, hip contact area for various loading configurations

	% Change relative to intact, hip contact area													
	Stance		Flexion		Extension		Right LB		Left LB		Right AR		Left AR	
Side	Right	Left	Right	Left	Right	Left	Right	Left	Right	Left	Right	Left	Right	Left
Unilateral—L	—	—	9.48	3.60	−1.26	—	−0.42	—	2.25	−8.98	−1.34	—	—	0.97
Unilateral—R	5.36	—	2.59	8.56	−5.44	—	2.52	0.50	−1.35	—	6.70	4.35	—	—
Bilateral	5.36	—	2.59	2.70	−3.77	—	2.52	−2.99	—	−3.67	8.93	—	3.57	—

*Notes*: Negative values = decrease relative to intact; (−) = no change relative to intact.

Abbreviations: AR, axial rotation; L, left; LB, lateral bending; R, right.

## 4 DISCUSSION

The current study sought to characterize stresses on the hip joint of a spine‐sacroiliac‐hip model before and after SI joint fixation to assess the potential development of ASD at the hip. To develop the SSIH, individual segment models were developed, validated, and then combined into a single model. During validation of the hip model, there was noted variability in the previously published contact area and stress data.^20^, ^22^ This variability may be a result of the method for calculating contact area (eg, experimental vs FEA, cutoff stress) or differing cartilage material properties. In a separate experimental study, Bay et al simulated single leg stance and measured mean hip contact pressures of ~4.5 MPa and average total contact areas of ~530 mm.^2^, ^32^ Although there is variability between previous studies, the contact areas and contact stresses for the current hip model are consistent with the reported range demonstrating that the hip segment model described here is valid and was applicable for use in the SSIH model.

The creation of the SSIH model and confirmation of its functionality was guided by the data governing the individual lumbopelvic and hip validations. To the authors' knowledge, such a model incorporating the spine, pelvis, and femora, with explicit definition of SI and hip joint function, is not currently described in the orthopedic research literature. While lack of an experimental model incorporating all of these elements is a limitation in the validation of the combined model, the lumbopelvic and hip validations described in this and previous^24^ research serve as reasonable comparisons in the justification of the combined model's function. As expected, SI joint ROM for the intact, combined model in double leg stance was less than that of the intact model in single leg stance and consistent with the double leg stance model described in the previous validation.^24^ Whereas the pelvic ring retains more flexibility in single leg stance, double leg stance results in further constraint of the motion (ie, more stability)^33^ at the pubic symphysis and SI joints, thereby resulting in smaller ROMs in the intact case. As for hip stress, magnitudes were not expected to exceed those determined in the validation studies as the position of the femora represented standing rather than femoral positions corresponding with more dynamic motions such as walking and ascending or descending stairs. Hip stress in the intact combined model was, in fact, found to be lower than that measured during verification and similar in magnitude to average stresses described by Harris et al.^20^ Given the above information, the functionality of the combined model was confirmed, thereby supporting the use of this model to study the effects of SI joint treatment on the hip. Further, the current model could be expanded upon and used in future studies for the purpose of investigating the effects of functional changes in one joint on another.

Similar to what previous studies have shown,^13^ treating the SI joint of the SSIH model either unilaterally or bilaterally with triangular implants resulted in a reduction in SI joint motion in comparison to the intact case. Unilateral treatments resulted in a greater reduction in motion of the treated side as compared to the contralateral, intact side, a finding that is in agreement with other studies.^13^, ^14^, ^34^ Compared with unilateral treatment, bilateral treatments resulted in minimal reductions in motion of the primary SI joint, yet another result supporting previous findings.^13^ Interestingly, some asymmetry was noted in the model, which was evident from the differences seen between the left and right sides during flexion and extension; this was noted in both the intact and bilateral cases. Despite this, the trend in reductions and similar magnitudes were representative of previous work, which further supports the use of this model to glean additional information, not only about the SI joint, but about how it affects surrounding joints as well.

Specifically, this model was used to assess the effects of SI joint fixation on the hip to address whether the hip may be at risk of degeneration. Average and peak stresses measured in the SSIH model were similar in magnitude to those reported by Harris et al.^20^ Further, the literature supports the peak stress magnitudes found here as maximum pressures of 5 MPa to as high as 18 MPa have been reported.^33^, ^35^ Following treatment, generally low changes (10% or less) in stress were observed at each hip joint (Figure 9). Changes greater than 10% mostly accounted for decreases in stress, while a few instances accounted for increases; however, these increases equated to magnitudes totaling less than 2 MPa, appreciably lower than the previously reported peak stress magnitudes.

With regard to contact area, a slightly higher area was measured for the right hip than the left in stance and bilateral treatment. These differences suggest that the previously noted asymmetry was likely due to an anatomical difference between left and right sides of the pelvis.^36^ Overall, changes in contact area relative to intact were less than 10% regardless of treatment; this was true for both right and left hip joints. Finally, contour plots depicting the contact area for a given motion before and after treatment were observed; stress patterns were considered reasonable when compared to the literature.^20^, ^22^ While definitive clinical commentary cannot be made regarding the development of hip degeneration following SI joint stabilization with triangular titanium implants, the above results suggest minimal changes in contact stress and area occur at the joint; and therefore, the potential for developing hip joint degeneration is likely low.

Some limitations are present in this study. First, the femora of the model originated from a different CT scan than the spine and pelvis. The lumbopelvic model used in this study was sourced from an earlier study and the specimen did not include femora at the time of the scan; thus, femora were sourced from a second scan. However, to lessen the impact of the difference in specimens, the models were both generated from female donors. Further, to accommodate potential anatomical differences in fit between the acetabulae and femora of the two specimens, the femora were scaled and aligned relative to the pelvis per Wu et al.^25^ Interference between the femoral head and acetabulum for each hip joint was assessed prior to finalizing the model such that the joint space was representative of data found in the literature.^26^

A second limitation of the model was the representation of hip musculature as connector elements for the purpose of simplifying the overall model. Though the muscles were characterized by passive connector elements rather than active elements equating to muscle contraction, they sufficiently supported the combined model during the applied physiologic loading. Further, the connector elements were attached to the model at physiologic origin and insertion points, which ensured that they were acting at realistic locations on the model. Despite the simplification, the model allowed hip motion and thus represented an improvement over other finite element models described in the literature, which included fixed acetabulae or immobile femora.^13^, ^24^, ^37^ Future studies could expand upon the current model by incorporating active musculature as a physiologic constraint.

Finally, this study utilized double leg stance with a moment applied at the spine of a single model; other motions or loading configurations were not examined here. While “worst case” movements demonstrating impact such as running or jumping were not modeled, a stance model with applied moment was considered representative of day‐to‐day demands on the body. Further, while different test protocols exist, (ie, specifically load‐control, displacement control, or a hybrid protocol), a load‐control approach better demonstrates the post‐operative motion of a patient in comparison to the displacement and hybrid control methods.^38^ Additionally, in a previous study, little difference (6%) was demonstrated in the applied bending moment^14^ when using the hybrid protocol to evaluate SI joint treatment via triangular titanium implants. As the difference in loading was minimal, a load control (moment with follower load) approach was adopted here. Regardless of the test protocol, the current work presents a multi‐segment model with mobile hip joints that allows for the investigation and, moreover, quantification of hip contact stress and area, which is a new and useful contribution to the field. Future studies could expand upon this work by increasing the number of modeled specimens and/or modeling the hip joint in postures other than stance.

The current research demonstrated the effects of SI joint stabilization on the hip joint. Utilizing a previously developed model that combined the spine, pelvis, and femora, SI joint ranges of motion and stresses and contact area at the hip joint were evaluated following unilateral (left or right) and bilateral treatment of the SI joint with triangular titanium implants. Relative to the intact condition, SI joint ROMs were reasonable and changes in stress were mostly minimal; all stress magnitudes were within reported ranges for hip cartilage. Contact area also demonstrated small changes following SI joint fixation. While computational studies alone cannot be used to make definitive clinical conclusions, the outcome of the current work suggests that the risk of developing adjacent segment disease at the hip joint following SI joint stabilization is likely low. Further studies may be conducted to assess patient outcomes and clinically confirm the results of this computational study.

## CONFLICT OF INTEREST

R. Chande, D. Lindsey, and S. Yerby are employees of and have stock options/stock in SI‐BONE; R. D. Carpenter and B. Duhon are consultants for SI‐BONE.

## AUTHOR CONTRIBUTIONS

All authors have read and approved the final submitted manuscript. A.J. created the FE models, acquired the data, and advised on details of the manuscript. R.C. advised on model development, analyzed the data, and drafted the manuscript. R.D.C. assisted with model development and data analysis. D.L. assisted with model development and data analysis, and edited the manuscript. D.E. provided feedback regarding model development and assisted with data acquisition. S.Y. advised on model development and edited the manuscript. B.D. advised on project concept and provided clinical feedback for the research. V.G. advised on model development and data analysis.
